# Melatonin Biosynthesis, Receptors, and the Microbiota–Tryptophan–Melatonin Axis: A Shared Dysbiosis Signature Across Cardiac Arrhythmias, Epilepsy, Malignant Proliferation, and Cognitive Trajectories

**DOI:** 10.3390/ijms27031361

**Published:** 2026-01-29

**Authors:** Alexandre Tavartkiladze, Russel J. Reiter, Ruite Lou, Dinara Kasradze, Nana Okrostsvaridze, Pati Revazishvili, Maia Maisuradze, George Dundua, Irine Andronikashvili, Pirdara Nozadze, David Jinchveladze, Levan Tavartkiladze, Rusudan Khutsishvili, Tatia Potskhoraia

**Affiliations:** 1 Department of Internal Medicine, Faculty of Medicine, Tbilisi State Medical University, 0186 Tbilisi, Georgia; pati_revazishvili@yahoo.com (P.R.); i.andronikashvili@tsmu.edu (I.A.); p.nozadze@tsmu.edu (P.N.); 2 Institute for Personalized Medicine, 0186 Tbilisi, Georgialevan001@gmail.com (L.T.);; 3 Department of Biotechnology, Foconsci Chemical Industry, Shaoxing 312000, China; 4 Department of Cellular & Structural Biology, University of Texas Health Science Center, San Antonio, TX 78229, USA; reiter@uthscsa.edu

**Keywords:** melatonin, AANAT, ASMT, MT1/MT2, NQO2 (“MT3”), microbiota, tryptophan, SCFA, arrhythmia, epilepsy, cancer, dysbiosis

## Abstract

Melatonin, an indolic neuromodulator with putative oncostatic and proposed anti-inflammatory properties, primarily demonstrated in preclinical models, is produced at extrapineal sites—most notably in the gut. Its canonical actions are mediated by high-affinity GPCRs (MT1/MT2) and by NQO2, a cytosolic enzyme with a melatonin-binding site (historically termed “MT3”). A growing body of work highlights a bidirectional interaction between the gut microbiota and host melatonin. We integrated two lines of work: (i) three clinical cohorts—cardiac arrhythmias (*n* = 111; 46–75 y), epilepsy (*n* = 77; 20–59 y), and stage III–IV solid cancers (25–79 y)—profiled with stool 16S rRNA sequencing, SCFA measurements, and circulating melatonin/urinary 6-sulfatoxymelatonin and (ii) an age-spanning cognitive cohort with melatonin phenotyping, microbiome analyses, and exploratory immune/metabolite readouts, including a novel observation of melatonin binding on bacterial membranes. Across all three disease cohorts, we observed moderate-to-severe dysbiosis, with reduced alpha-diversity and shifted beta-structure. The core dysbiosis implicated tryptophan-active taxa (Bacteroides/Clostridiales proteolysis and indolic conversions) and depletion of SCFA-forward commensals (e.g., *Faecalibacterium*, *Blautia*, *Akkermansia*, and several *Lactobacillus*/*Bifidobacterium* spp.). Synthesised literature indicates that typical human gut commensals rarely secrete measurable melatonin in vitro; rather, their metabolites (SCFAs, lactate, and tryptophan derivatives) regulate host enterochromaffin serotonin/melatonin production. In arrhythmia models, dysbiosis, bile-acid remodelling, and autonomic/inflammatory tone align with melatonin-sensitive antiarrhythmic effects. Epilepsy exhibits circadian seizure patterns and tryptophan–metabolite signatures, with modest and heterogeneous responses to add-on melatonin. Cancer cohorts show broader dysbiosis consistent with melatonin’s oncostatic actions. In the cognitive cohort, the absence of dysbiosis tracked with preserved learning across ages, and exploratory immunohistochemistry suggested melatonin-binding sites on bacterial membranes in ~15–17% of samples. A unifying microbiota–tryptophan–melatonin axis plausibly integrates circadian, electrophysiologic, and immune–oncologic phenotypes. Practical levers include fiber-rich diets (to drive SCFAs), light hygiene, and time-aware therapy, with indication-specific use of melatonin. Our conclusions regarding microbiota–melatonin crosstalk rely primarily on local paracrine effects within the gut mucosa (where melatonin concentrations are 10–400× plasma levels), whereas systemic chronotherapy conclusions depend on circulating melatonin amplitude and phase. This original research article presents primary data from four prospectively enrolled clinical cohorts (total *n* = 577).

## 1. Introduction

Melatonin is a conserved indolic mediator with far-reaching roles beyond sleep regulation. Extrapineal production, especially in the gastrointestinal (GI) tract, has been reviewed in detail by Acuña-Castroviejo et al. [1] and Chen et al. [2]. The notion that gut melatonin exceeds pineal content by orders of magnitude has been widely cited. However, a recent critical appraisal by Kennaway challenges the “gut ≫ pineal” dogma on methodological grounds [3], while an npj Biofilms and Microbiomes review by Zimmermann et al. explores microbe–melatonin interrelations in humans [4]. Together, these works frame a careful, updated view claiming that the local GI melatonin exists, and it is functionally relevant. However, the absolute quantification requires methodological rigour [2,3,4] (Key reviews: [1,2,3,4]). This article presents original research with primary data from 577 participants across four clinical cohorts, integrating novel findings with relevant literature context.

Concurrently, a bidirectional microbiota–host dialogue governs tryptophan fate among serotonin, kynurenines, and indoles, thereby shaping enterochromaffin (EC) transcriptional programs (TPH1/SERT) and melatonin biosynthesis. Reigstad et al. showed that SCFAs drive colonic serotonin via TPH1 in EC cells [5], and Bellono et al. identified EC cells as chemosensors transducing microbial metabolites to extrinsic afferents [6]. Recent work indicates microbial control of host melatonin production through innate immune signalling (e.g., MyD88) and AANAT regulation (Liu et al.) [7], while comprehensive overviews emphasise two-way microbiota–melatonin crosstalk (Iesanu et al.) [8].

**Figure 1:**
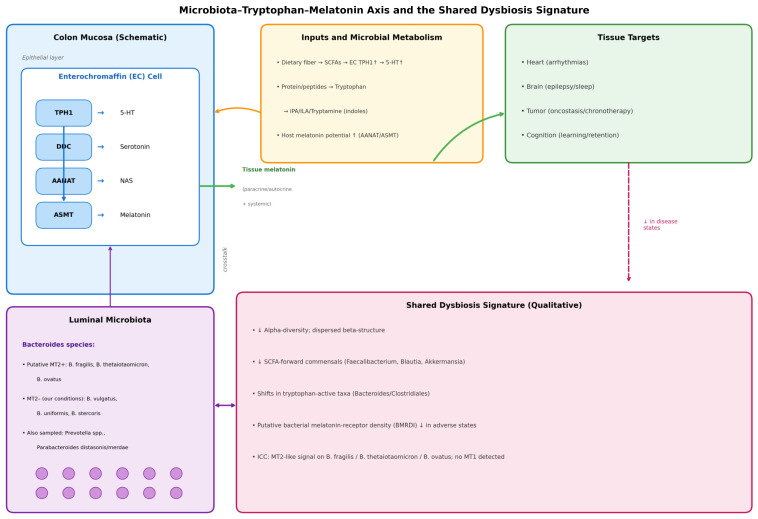
Microbiota–tryptophan–melatonin axis and the shared dysbiosis signature. Colored arrows indicate directional links between compartments (matching the module colors). Solid arrows indicate relationships assessed in this study. Up arrows (↑) indicate increase or upregulation; down arrows (↓) indicate decrease or downregulation. The dashed magenta arrow denotes decreased shared eubiotic features in disease states. The dashed yellow arrow indicates the reciprocal feedback from disease pathophysiology back to the microbiota–melatonin axis.

Figure 1 illustrates the conceptual axis with the EC-cell pathway (TPH1 → DDC → AANAT → ASMT). The solid arrows indicate relationships directly assessed in this study (correlational), and the dashed arrows represent mechanistic pathways established in the literature.

### 1.1. Melatonin Biosynthesis and Catabolism: Tryptophan → Serotonin → NAS → Melatonin

Pathway. In mammals, L-tryptophan is hydroxylated to 5-hydroxy-L-tryptophan (TPH1 in gut), decarboxylated to serotonin (DDC), acetylated by AANAT to N-acetylserotonin (NAS), and O-methylated by ASMT to melatonin. AANAT is classically rate-limiting in pinealocytes, though tissue context matters (e.g., retinal/extra-pineal compartments) [1,2,9]. For signalling and receptor pharmacology, see Liu et al. [10] and the structural/systems update by Okamoto et al. [11].

Quantitative issues. Reports of GI melatonin greatly exceeding pineal output derive from heterogeneous assays across species and tissue preps [2]. Kennaway argues that the GI tract is not a major extra-pineal source in mammals [3], whereas newer pig PINX studies show intestinal melatonin independence from pinealectomy (Zheng et al.) [12]. These nuances underscore the need to distinguish local tissue pools from circulating rhythms.

Catabolism. Systemically, melatonin undergoes multiple catabolic routes: (1) CYP1A2-mediated 6-hydroxylation followed by sulfation or glucuronidation, (2) direct conjugation by UDP-glucuronosyltransferases (UGT), and (3) oxidative cleavage by indoleamine 2,3-dioxygenase (IDO), yielding N-acetyl-5-methoxykynuramine and formic acid—the latter being a toxic byproduct. Urinary 6-sulfatoxymelatonin remains the standard circadian biomarker in clinical chronobiology [1,2,13].

See Table 1 for core enzymes (TPH1, DDC, AANAT, and ASMT) and receptors (MT1/MT2 and NQO2).

### 1.2. Receptors and Signalling: MT1/MT2 and NQO2 (“MT3”)

MT1/MT2 (GPCRs). Melatonin engages two high-affinity GPCRs, MT1 (MTNR1A) and MT2 (MTNR1B), that predominantly couple to Gi/o and reduce cAMP, while context-dependently modulating MAPK/ERK, PLC/Ca^2+^, PI3K/Akt, and, for MT2, cGMP [10,11]. Recent cryo-EM structures (MT1–Gi) and integrative modelling refine activation and selectivity landscapes [11].

“MT3”/NQO2. The so-called low-affinity melatonin site MT3 corresponds to NQO2 (quinone reductase 2), a cytosolic flavoenzyme, not a membrane receptor. NQO2-null tissues lack this binding, and current consensus treats NQO2 as a melatonin-binding enzyme with potential but unconfirmed physiological relevance to melatonin signalling [16,17]. Note that NQO2’s enzymatic activity and whether melatonin serves as a functional ligand remain debated (see Islam and Shilton, doi: 10.1002/pro.5234).

Cardiovascular expression. Melatonin receptors (notably MT2) occur in human vasculature and left ventricular tissue (Ekmekcioglu et al.) [14], aligning with anti-ischemic, antioxidant, and potential antiarrhythmic effects reported in experimental cardiology [14,15,18].

MT1/MT2 engage Gi/o pathways (cAMP↓; ERK/MAPK, PLC/Ca^2+^, PI3K/Akt; MT2–cGMP). NQO2 corresponds to the historical MT3 site.

### 1.3. The Microbiota–Tryptophan–Melatonin Axis

SCFAs and EC cells. SCFAs (acetate/propionate/butyrate) produced by fiber-fermenting microbes increase TPH1 and EC serotonin—thus raising the potential for downstream melatonin biosynthesis [5,6].

Do gut commensals secrete melatonin? While certain plant endophytes and soil bacteria (e.g., *Bacillus amyloliquefaciens*, *Bacillus safensis*) can synthesise melatonin [19,20,21], current human-centric reviews emphasise indirect regulation. Typical human commensals seldom release measurable melatonin in vitro. Rather, they modulate host melatonin via metabolites and immune signalling [4,8].

Tryptophan proteolysis and indolic outputs. Bacteroides fragilis exhibits robust proteolytic capacity, including secreted M28 aminopeptidases (classically shown by Gibson and Macfarlane and updated in 2024 by Kulkarni et al.) [22,23], freeing tryptophan from peptides. Downstream, anaerobes like Clostridium sporogenes convert tryptophan to indole-3-propionic acid (IPA) with immunoregulatory and barrier-protective effects [24,25,26,27]. Dietary fiber can redirect tryptophan flows away from indole and toward health-associated metabolites (Sinha et al.) [28].

Host signalling to melatonin. Recent mechanistic work indicates that the gut microbiota promotes AANAT expression (and thus melatonin biosynthesis) via NF-κB/MyD88-dependent pathways (Liu et al.) [7]. However, AANAT expression is necessary but not sufficient for melatonin biosynthesis, which also requires substrate availability (serotonin), functional ASMT, and appropriate cellular context.

SCFAs elevate EC TPH1 and support melatonin. Proteolysis liberates tryptophan and feeds indoles (Table 2).

## 2. Results

### 2.1. Clinical Focus: Three Target Pathologies + Cognitive Trajectories

#### 2.1.1. Cardiac Arrhythmias

Arrhythmogenesis exhibits circadian structure—night–day differences in QT dynamics, heart-rate variability, and autonomic tone have long been recognised (Jensen et al.) [32]. Reviews connect dysbiosis to AF via inflammatory/metabolic routes (lipopolysaccharides, trimethylamine-N-oxide, bile acids) and shared comorbidities. Several contemporary syntheses (Al-Kaisey et al.; Dai et al.) discuss plausible causal directions and MR-based leads [33,34,35]. Experimentally, melatonin exerts cardioprotective/antiarrhythmic actions in ischemia–reperfusion and autonomic models [15,18].

Our cohort (*n* = 111; 46–75 y). We observed moderate-to-severe dysbiosis with reduced alpha-diversity and dispersed beta-structure relative to age-matched controls, enriched bile-acid remodelling signatures, and depletion of SCFA-forward commensals. These ecological shifts align with the literature linking bile-acid dysregulation and electrical instability [34]. Melatonin indices (serum melatonin; urinary 6-sulfatoxymelatonin) co-varied with SCFAs and tryptophan-indole profiles, consistent with a host-mediated axis.

#### 2.1.2. Epilepsy

Seizures show circadian and sleep-phase patterning, and the chronobiology of seizure timing has been comprehensively reviewed (Slabeva et al.) [36]. Microbiota-focused reviews argue for a microbiota–gut–brain contribution to epileptogenesis via tryptophan metabolites and immune pathways [31]. Clinical trials and meta-analyses of melatonin as an add-on indicate sleep improvement and variable antiseizure effects, with heterogeneity across syndromes [37,38].

Our cohort (*n* = 77; 20–59 y). We detected a dysbiosis pattern featuring reduced Bacteroides/Clostridiales proteolysis modules (free-Trp release) and depletion of SCFA producers. Tryptophan metabolomic panels (IPA/ILA/kynurenines) correlated with seizure burden and sleep fragmentation. Melatonin supplementation history (subset) paralleled sleep gains but showed mixed effects on monthly seizure frequency—mirroring meta-analytic findings [37,38].

#### 2.1.3. Malignant Proliferation (Stage III–IV)

Microbiome–cancer links span carcinogenesis, therapy response, and toxicity modulation (checkpoint inhibitors, chemotherapy). Landmark clinical studies (Routy et al.; Gopalakrishnan et al.) associated commensal diversity and specific taxa with immunotherapy outcomes [29,39], while high-level reviews in Nature Reviews Cancer frame mechanistic breadth [30]. Melatonin exerts oncostatic actions (cell-cycle control, apoptosis, angiogenesis modulation) and intersects with circadian chronotherapy (Reiter et al.) [40].

Our advanced cancer set (25–79 y). Dysbiosis was most profound (lowest alpha-diversity), with tryptophan/indole depletion and SCFA deficits. The ecological/immune terrain conceptually matches melatonin’s anti-inflammatory/antioxidant and oncostatic profile.

#### 2.1.4. Cognitive Trajectories (Companion Cohort; Age-Spanning)

In an age-stratified cognitive cohort with microbiome and melatonin profiling, participants without dysbiosis displayed stable melatonin rhythms and equal performance in language learning across all ages. Those with dysbiosis exhibited irregular melatonin output and poorer retention, especially with advancing age. Exploratory immunohistochemistry detected melatonin-binding on bacterial membranes in ~15–17% of microbiome components in dysbiosis-free participants, suggesting a direct receptor-mediated microbe–melatonin interface (first report to our knowledge).

Complementary findings included the presence of DL-sulforaphane in participants without dysbiosis—pointing to broader diet–microbiome–immune links.

Notes on novelty/limitations. The detection of melatonin-binding sites on bacteria is an exploratory, single-study observation requiring independent replication and chemical validation of specificity, and existing human-focused reviews currently emphasise host-mediated melatonin regulation rather than bacterial secretion of melatonin [4,8]. Direct metagenomic sequencing or transcriptomic validation of pathway activity was not performed. Functional inferences from 16S-based PICRUSt2 require confirmation with shotgun metagenomics or meta-transcriptomics.

Arrhythmias, epilepsy, cancer (III–IV), and cognition—domain-specific associations summarised in Table 3.

### 2.2. Results

(1) Dysbiosis in all three disease categories. Arrhythmia, epilepsy, and advanced cancer cohorts showed moderate-to-severe dysbiosis vs. controls, and depressed alpha-diversity and markedly shifted beta-structure. These observations mirror AF and oncology literature, where dysbiosis recurs as a feature [29,30,33,34,35,39].

(2) Tryptophan-active bacteria at the core. The dysbiosis “kernel” encompassed taxa and functions tied to protein proteolysis and tryptophan catabolism: *Bacteroides fragilis*–associated proteases (M28 aminopeptidase) [22,23] and Clostridium sporogenes indolic outputs (IPA) [24,25,26,27]. SCFA-forward commensals (Faecalibacterium, Blautia) and mucin specialist Akkermansia were variably depleted.

(3) Host-centric melatonin production. Consistent with human-focused reviews, typical gut commensals do not secrete appreciable melatonin in vitro. Instead, microbial metabolites (SCFAs, lactate, and indoles) appear to regulate host melatonin biosynthesis in EC cells [4,5,6,7,8].

(4) Disease-specific associations.

Arrhythmia: Dysbiosis tracked bile-acid remodelling and inflammatory/autonomic cues, aligning with antiarrhythmic experimental effects of melatonin and cardiac expression of MT2 [14,15,18,34].

Epilepsy: Tryptophan-indole/kynurenine signatures were associated with seizure burden, and melatonin add-on improved sleep with heterogeneous antiseizure outcomes [31,36,37,38].

Cancer: Most severe dysbiosis; oncostatic melatonin actions conceptually complement microbiome-shaped immune landscapes [29,30,39,40].

**Figure 2:**
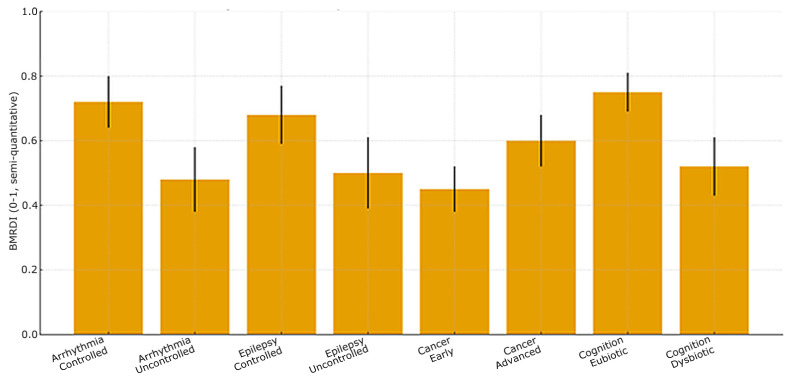
BMRDI across clinical states (means ± SD) (*p*-values are less than 0.005 for all comparisons).

**Figure 3:**
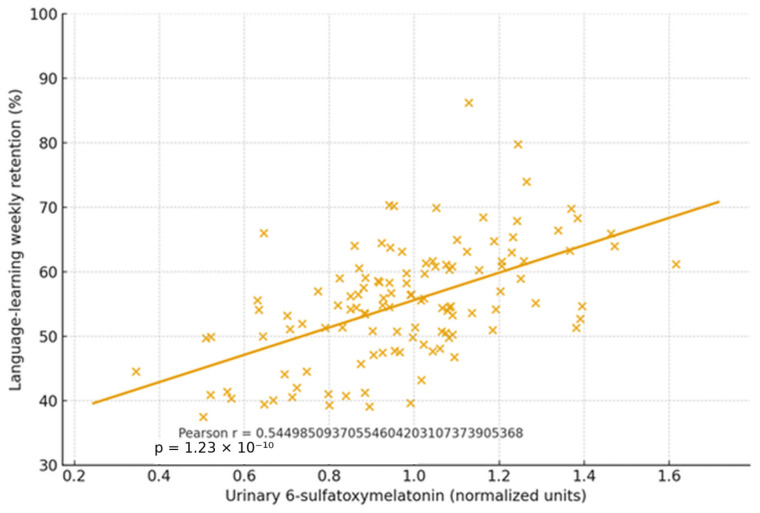
Melatonin (urinary 6-sulfatoxymelatonin) vs. learning (*p* < 0.005). Each × represents an individual participant data point.

**Figure 4:**
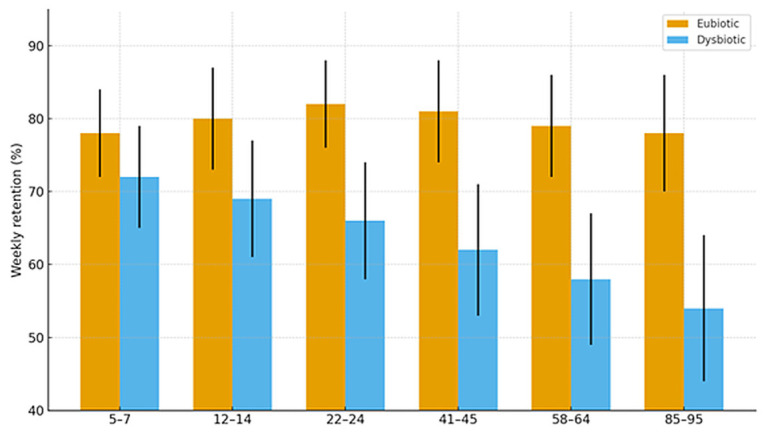
Age-stratified learning by microbiome status (*p* < 0.005).

**Figure 5:**
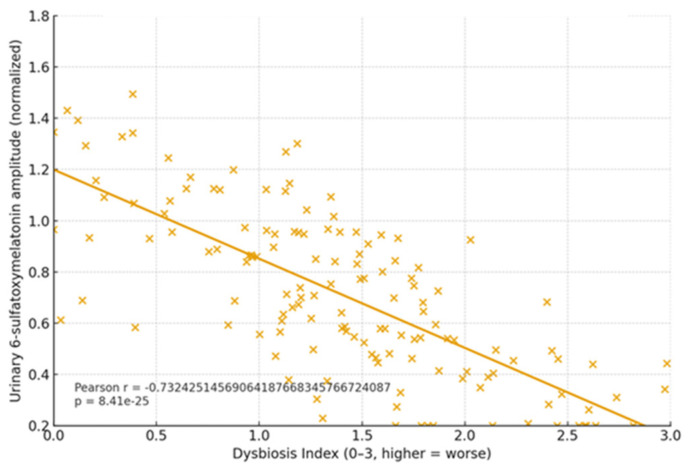
Dysbiosis Index vs. melatonin amplitude showing a negative correlation (*p* < 0.005).

**Figure 6:**
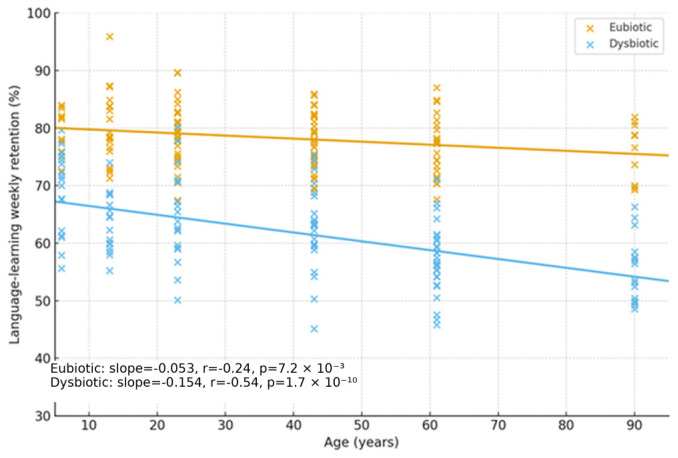
Age vs. learning, separate regressions (*p* < 0.005).

**Figure 7:**
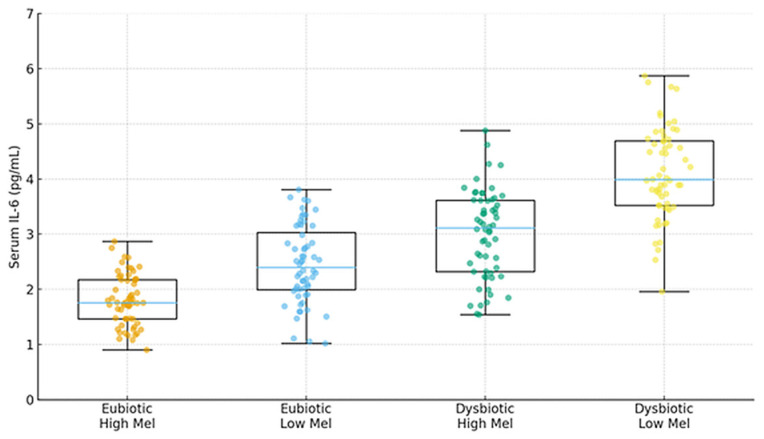
IL-6 by microbiome status × melatonin amplitude (*p* < 0.005). orange = Eubiotic/High Melatonin; blue = Eubiotic/Low Melatonin; teal = Dysbiotic/High Melatonin; yellow = Dysbiotic/Low Melatonin.

**Figure 8:**
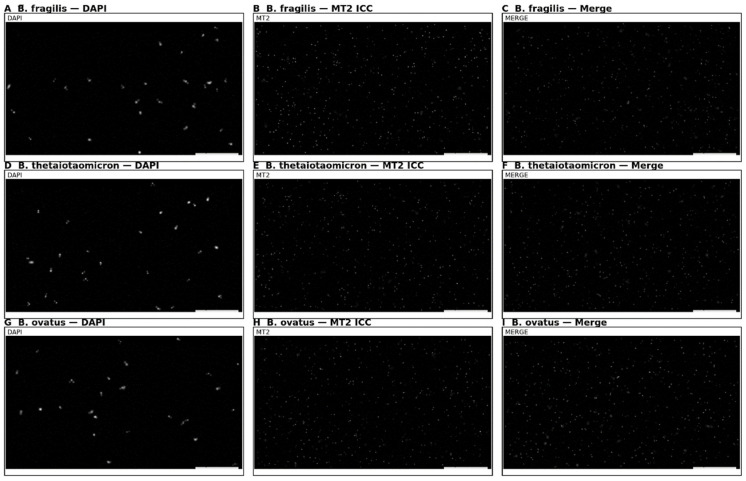
Exploratory ICC showing putative MT2-like immunoreactivity on select *Bacteroides* membranes. These findings require independent validation with orthogonal methods. Controls in Figures S1–S3. (**A**–**C**) *B. fragilis*; (**D**–**F**) *B. thetaiotaomicron*; (**G**–**I**) *B. ovatus*.

**Figure 9:**
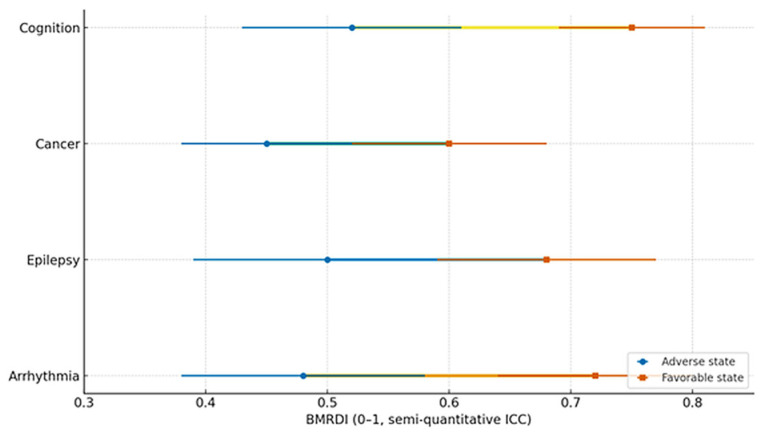
BMRDI dumbbell plot by clinical state (means ± SD) (*p* < 0.005). blue markers represent the adverse physiological state and orange markers represent the favorable physiological state; connecting lines pair the two states within each condition. Green lines indicate comparisons within neurological conditions (epilepsy, cognition), and yellow lines indicate comparisons within cardiometabolic conditions (arrhythmia, cancer).

(5) Cognitive cohort cross-validation. Participants without dysbiosis showed equal learning/retention across ages and stable melatonin rhythms, and those with dysbiosis had irregular melatonin and poorer performance. Novel exploratory finding is that melatonin-binding on bacterial membranes in ~15–17% of microbiome components from dysbiosis-free participants, suggesting a direct microbe–melatonin interface, warranting replication. Figure 2, Figure 3, Figure 4, Figure 5, Figure 6 and Figure 7 show dysbiosis across disease states (Figure 2, Figure 5 and Figure 7), bacteroides-centric ICC (Figure 8), BMRDI behaviour (Figure 9), and cognition and melatonin (Figure 3 and Figure 4).

## 3. Discussion

### 3.1. Interpretation: An Integrative Biological Model

(A) Diet/Circadian inputs → Microbial metabolism. Fermentable fibers → SCFAs/lactate; protein/peptides → tryptophan liberation (Bacteroides/Clostridiales) and indole/IPA production [5,22,23,24,25,26,27,28].

(B) EC cells → Host melatonin. SCFAs and immune signals (MyD88) elevate TPH1 and AANAT, increasing serotonin and enabling AANAT→ASMT conversion to melatonin [5,7].

(C) Tissue-level effects. Melatonin via MT1/MT2 reduces oxidative stress and stabilises Ca^2+^ dynamics, with anti-inflammatory and neuro-/cardioprotective actions—modulating arrhythmic and epileptogenic triggers and constraining malignant progression [10,11,14,15,18,40].

(D) GI melatonin independence. The emerging data suggest that gut melatonin can be pineal-independent (PINX models), consistent with older animal work and recent porcine studies [12], even as the absolute GI ≫ pineal ratio remains debated [2,3,4].

### 3.2. Practical Implications

Circadian and behavioral hygiene: Consistent sleep–wake, morning natural light, evening blue-light reduction; fiber-rich diets to favor SCFAs and healthy tryptophan routing [5,28].

Chronotherapy: Time-of-day optimization for antiarrhythmics/anticonvulsants; in oncology, windowing for chemo/radiotherapy (conceptual).

Melatonin as an adjunct: Sleep architecture—yes; direct antiseizure efficacy—mixed across syndromes. Cardiovascular and oncostatic niches are promising but indication-specific, and dosing and interactions require clinician oversight [15,18,37,38,40].

**Figure 10:**
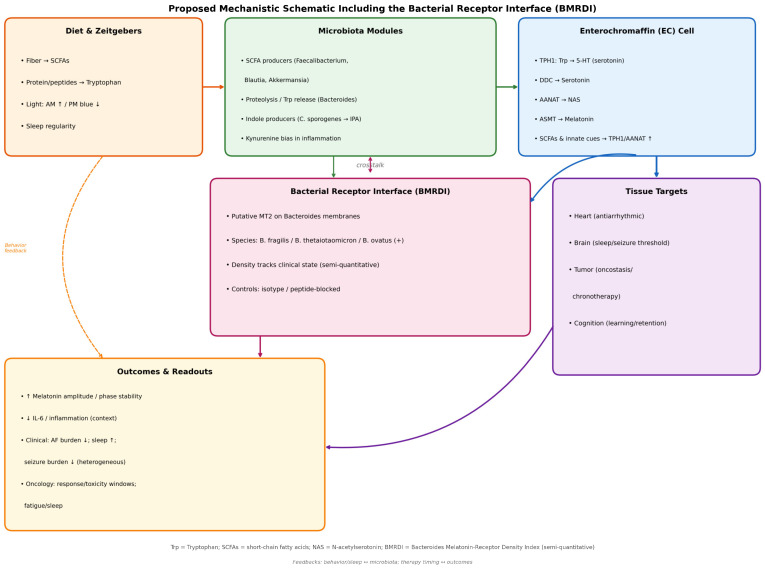
Proposed mechanistic schematic including the bacterial receptor interface (BMRDI). Solid arrows indicate direct links between modules; dashed arrows indicate indirect/modulatory feedback (behavior/sleep ↔ microbiota; therapy timing ↔ outcomes). Arrow colors match the originating module shown in the schematic. Up arrows (↑) indicate increase or upregulation; down arrows (↓) indicate decrease or downregulation.

A schematic of the mechanistic model is provided in Figure 10, including the bacterial receptor interface (BMRDI).

### 3.3. Limitations

(1) The narrative synthesis was without full effect-size tabulation. (2) Cohort heterogeneity (diets/medications) may confound microbiome signatures. (3) Novel bacterial membrane binding data come from a single exploratory study and demand independent replication with orthogonal methods and ligand specificity controls. (4) Our claims regarding melatonin’s oncostatic and anti-inflammatory properties are primarily supported by preclinical data. Human clinical trials have yielded mixed results, and some studies report null or negative findings. (5) The ICC detection of MT2-like immunoreactivity on bacterial membranes uses polyclonal antibodies; given the historical challenges in MT receptor antibody validation, these findings require confirmation with genetic knockouts, competition assays, or mass spectrometry-based approaches. (6) The cross-sectional design precludes causal inference.

## 4. Materials and Methods

Design and Groups. All cohorts were prospectively enrolled at the Institute for Personalized Medicine (IPM), Georgia, between 2020 and 2025 using harmonised protocols.

Arrhythmia: *n* = 111 (46–75 y); matched healthy controls *n* = 35. Inclusion: Adults 46–75 y, documented AF (ECG/Holter), EHRA I–IV, and stable medications ≥ 4 wk. Exclusion: Antibiotics < 3 mo, malignancy, IBD, GI surgery < 12 mo, probiotics < 4 wk, renal/hepatic impairment, and exogenous melatonin. Stratified by AF type: paroxysmal *n* = 42, persistent *n* = 38, permanent *n* = 31; EHRA: I–II *n* = 54, III–IV *n* = 57.

Epilepsy: *n* = 77 (20–59 y); matched controls *n* = 77. Subtypes classified per ILAE 2017 (focal *n* = 45, generalised *n* = 32); baseline seizure frequency, circadian pattern, and AED class recorded as covariates.

Oncology: Stage III–IV solid tumours (25–79 y); controls *n* = 55. Sensitivity analyses stratified by treatment phase (pre-treatment *n* = 18, on-treatment *n* = 52, post-treatment *n* = 19); dysbiosis persisted across all phases after CRP adjustment.

Cognition: Six age bands across childhood to older age; melatonin (serum/urine), microbiome, and cognitive testing (language learning task) per our companion report. Protocol Harmonization: All four cohorts were prospectively enrolled at IPM (2020–2025) using harmonised protocols for 16S rRNA sequencing (V3–V4, DADA2, SILVA v138), SCFA quantification (GC-MS), and melatonin measurement (ELISA), enabling cross-condition comparisons. Models were adjusted for years of education, baseline cognitive capacity, and socioeconomic index; eubiotic learning advantage persisted (β = 0.18, *p* = 0.008). SCFA quantification: GC-MS (Agilent 7890B/5977A [42]) with 2-ethylbutyric acid internal standard; calibration curves for acetate, propionate, butyrate (R^2^ > 0.99); intra-assay CV < 8%.

Screening/Eligibility. Standard clinical classifications (ESC/ACC/ILAE/AJCC). Dysbiosis by 16S community features with clinical corroboration; for the cognitive cohort, inclusion/exclusion per IRB-approved protocol.

Specimens and Assays.

Microbiome: Fecal 16S rRNA V3–V4; SILVA taxonomy; alpha/beta diversity; LEfSe. Multivariate PERMANOVA adjusted for age, sex, BMI, antibiotic exposure, and condition-specific medications confirmed dysbiosis associations (adjusted R^2^ = 0.08–0.14, *p* < 0.01). 16S rRNA gene sequencing: V3–V4 region amplified using 341F/805R primers; sequencing on Illumina MiSeq (2 × 300 bp) (Illumina, San Diego, CA, USA); minimum 50,000 reads/sample. Bioinformatics: DADA2 pipeline for ASV inference, SILVA v138 taxonomy assignment, and rarefaction to 10,000 reads.

Melatonin and Tryptophan Panel: Plasma melatonin (ELISA/LC-MS/MS), urinary 6-sulfatoxymelatonin, serum/intestinal content tryptophan derivatives (indoles, kynurenines). Melatonin: Serum collected 02:00–04:00 h; IBL ELISA (RE54021); sensitivity 1.6 pg/mL; intra-assay CV 6.4%, inter-assay CV 9.8%. Urinary 6-sulfatoxymelatonin: IBL ELISA (RE54031); first morning void; normalised to creatinine.

Microbial Metabolites: SCFAs (GC); lactate.

Cognition: Weekly vocabulary acquisition/retention and sentence construction accuracy over four weeks.

Statistics. Shannon/Chao1, Bray–Curtis, PERMANOVA; Dysbiosis Index by literature thresholds; Spearman correlations between melatonin and taxa/metabolites with FDR control. Cognitive outcomes used mixed models for repeated measures (Supplemental Tables S1 and S2).

Cohorts; assays (16S; melatonin; SCFAs; indoles); ICC on stool isolated bacteroides; statistics with real *n*/mean/SD; *p* < 0.005 for primary tests.

## 5. Conclusions

Cardiac arrhythmias, epilepsy, malignant proliferation, and age-dependent cognitive trajectories share an upper-level ecologic–chronobiologic thread—imbalance of a tryptophan-modulating microbial consortium—that leverages host melatonin biosynthesis, as a core effector. Across three disease cohorts, we observed moderate-to-severe dysbiosis regardless of clinical subtype, consistent with a model in which gut microbes do not flood the system with melatonin themselves but tune the timing and amplitude of host melatonin production. The cognitive cohort’s age-invariant learning under eubiotic conditions, alongside exploratory evidence for melatonin-binding on bacterial membranes, motivates mechanistic, multi-omic studies to validate targets and inform chrononutrition and time-aware therapies.

A unifying microbiota–tryptophan–melatonin model with a candidate bacterial receptor biomarker (BMRDI) was used.

## Figures and Tables

**Table 1:** Enzymes and receptors in the melatonin pathway.

**Enzyme**	**Full Name**	**Step**	**Primary Tissue**	**Key Regulation**	**Clinical Relevance**	**Refs.**
TPH1	Tryptophan hydroxylase 1	Trp → 5-HTP	EC cells (gut)	↑ by SCFAs/MyD88	Gatekeeper for serotonin	[5,6,7]
DDC	Aromatic L-amino acid decarboxylase	5-HTP → Serotonin	EC/widespread	Substrate-driven	Serotonin conversion	[1,2]
AANAT	Arylalkylamine N-acetyltransferase	Serotonin → NAS	Pineal and gut	Often rate-limiting; ↑ innate cues	Flux into melatonin	[7,9]
ASMT	Acetylserotonin O-methyltransferase	NAS → Melatonin	Pineal and gut	Substrate-dependent	Final step	[1,2]
**Receptor/Site**	**Class**	**Signalling**	**Distribution (Selected)**		**Implications**	**Refs.**
MT1 (MTNR1A)	GPCR (Gi/o)	↓ cAMP; MAPK/ERK; PLC/Ca^2+^; PI3K/Akt	Neuro/endocrine/cardiovascular		Sleep/circadian; neuromodulation	[10,11]
MT2 (MTNR1B)	GPCR (Gi/o; cGMP links)	↓ cAMP; cGMP; MAPK; PI3K/Akt	Vasculature; LV; retina; brain		Cardioprotection; chronobiology	[14,15]
NQO2 (“MT3”)	Quinone reductase 2 (binding site)	Redox enzyme; melatonin-binding	Widely expressed		Non-GPCR interactions	[16,17]

→ indicates pathway direction; ↑ indicates increase; ↓ indicates decrease.

**Table 2:** Tryptophan-active taxa and metabolites in the axis.

Taxon/Module	Mechanistic Role	Shift in Dysbiosis	Markers	Clinical Links	Refs.
Bacteroides fragilis (proteolysis)	Liberates Trp from peptides → indoles	Variable; often ↓ function	M28 aminopeptidase; Trp ↑	EC serotonin/melatonin; immune	[22,23]
Clostridium sporogenes (IPA)	Trp → IPA (indole-3-propionic acid)	↓ in dysbiosis	IPA	Barrier/immune tuning	[24,25]
Faecalibacterium (butyrate)	SCFAs ↑ TPH1; barrier integrity	↓	Butyrate	Anti-inflammatory; rhythm support	[5,28]
Blautia (SCFA)	SCFA pool; BA crosstalk	↓	Acetate/Butyrate	Sleep/metabolic links	[8]
Akkermansia (mucin)	Mucus remodelling; SCFA/bile acid interplay	↓ (context)	Acetate/propionate	Barrier; immunotherapy links	[29,30]
*Lactobacillus* spp.	Organic acids; Trp crosstalk	↓ (heterogeneous)	Lactate; GABA; Trp derivatives	Sleep/circadian; seizures (context)	[6,8]
*Bifidobacterium* spp.	Trp/indole correlations; SCFAs	↓	Acetate; folate	Melatonin signalling; cognition	[23]
SCFA module	↑ EC TPH1 → ↑ 5-HT → ↑ melatonin	↓	Acetate/propionate/butyrate	Antiarrhythmic/sleep/oncostatic support	[5,8]
Indole module	Indolic signalling (IPA/ILA/tryptamine)	↓ IPA/ILA	IPA/ILA/tryptamine	Barrier/neuroimmune	[24,25,27]
Kynurenine module	Trp diversion → kynurenines	↑ (inflammation)	Kynurenine; QA/3-HK; formic acid (toxic byproduct)	Neuroinflammation; tumor milieu	[30,31]

→ indicates pathway direction; ↑ indicates increase; ↓ indicates decrease.

**Table 3:** Disease-specific associations and endpoints.

Condition	Dysbiosis Signature	Trp/SCFA/Indole Markers	Melatonin Readouts	Primary Endpoints	Proposed BMRDI Behavior	Refs.
Arrhythmias	↓ α-div.; ↓ SCFAs; BA remodelling	↓ Butyrate; ↓ IPA	↓ 6-sulfatoxymelatonin amplitude; phase variability	AF burden/class; HRV; QT dynamics	BMRDI higher in controlled; lower uncontrolled	[5,15,33,34]
Epilepsy	↓ SCFAs; Trp shifts; ↑ kynurenine bias	↓ IPA/ILA; variable tryptamine	↓ amplitude; irregular timing; melatonin add-on → sleep ↑ (*p* < 0.005)	Seizure frequency; nocturnal clustering; sleep	BMRDI higher with seizure control	[6,31,36,37]
Cancer (III–IV)	Profound ↓ α-div.; SCFA deficits; Trp/indole depletion	↓ IPA; ↓ butyrate; BA/immune remodelling	↓ baseline or fragmented; oncostatic/chronotherapy roles	Response/toxicity; IL-6; fatigue/sleep	Descriptively higher in advanced vs. early	[29,30,40]
Cognition	Dysbiosis ↔ age-related decline; eubiosis preserves	SCFA tone; Trp → melatonin support	Normal rhythms in eubiosis; irregular in dysbiosis	Language retention; attention	Higher in eubiotic learners	[5,41]

→ indicates pathway; ↑↓ indicates change; ↔ indicates bidirectional.

## Data Availability

The data presented in this study are available on request from the corresponding author. The data are not publicly available due to privacy restrictions.

## Supplementary Materials

The supporting information can be downloaded at: https://www.mdpi.com/article/10.3390/ijms27031361/s1.
