# Supplementary material for: Melatonin Biosynthesis, Receptors, and the Microbiota–Tryptophan–Melatonin Axis: A Shared Dysbiosis Signature Across Cardiac Arrhythmias, Epilepsy, Malignant Proliferation, and Cognitive Trajectories
Source: Int J Mol Sci. 2026 Jan 29;27(3):1361. doi: 10.3390/ijms27031361 (PMC12898085; doi:10.3390/ijms27031361)
Supplement: Supplementary file 1 [file ijms-27-01361-s001.zip › Supplementary_Materials.pdf]

Supplementary Materials

Melatonin Biosynthesis, Receptors, and the Microbiota–Tryptophan–Melatonin Axis:

A Shared Dysbiosis Signature across Cardiac Arrhythmias, Epilepsy, Malignant Proliferation, and Cognitive Trajectories

Alexandre Tavartkiladze et al.

Supplementary Figures: ICC Controls

The following supplementary figures provide validation controls for the immunocytochemistry (ICC) experiments detecting putative MT2-like immunoreactivity on bacterial membranes (Figure 8 in main text).

Figure S1. Isotype Control

Description: Isotype-matched control antibody (rabbit IgG) applied to *Bacteroides fragilis*, *B. thetaiotaomicron*, and *B. ovatus* preparations under identical conditions as MT2 primary antibody. Minimal background fluorescence confirms specificity of MT2 signal in Figure 8. Scale bar: 5 µm.

Figure S2. Peptide-Blocked Control

Description: MT2 primary antibody pre-incubated with blocking peptide (10× molar excess) before application to bacterial preparations. Abolition of signal demonstrates antibody specificity for the target epitope. Scale bar: 5 µm.

Figure S3. No-Primary Antibody Control

Description: Secondary antibody only (no primary antibody) applied to bacterial preparations. Absence of signal confirms that fluorescence in Figure 8 is not due to non-specific secondary antibody binding. Scale bar: 5 µm.

Table S1. Cohort Demographics Summary

Detailed demographic and clinical characteristics of the four study cohorts.

Characteristic	Arrhythmia (n=111)	Epilepsy (n=77)	Cancer (n=89)	Cognitive (n=300)
Age range (years)	46–75	20–59	25–79	5–95
Sex (M/F)	58/53	41/36	52/37	142/158
Disease classification	EHRA I–IV	ILAE 2017	TNM III–IV	N/A
Recruitment period	2020–2023	2020–2024	2020–2024	2021–2025
Setting	IPM Clinic	IPM Clinic	IPM Clinic	Community

Abbreviations: EHRA, European Heart Rhythm Association symptom scale; ILAE, International League Against Epilepsy; TNM, tumor-node-metastasis staging; IPM, Institute for Personalized Medicine.

Table S2. ICC Protocol Details

Parameter	Details
Primary antibody	Rabbit polyclonal anti-MT2 (MTNR1B), 1:200
Secondary antibody	Goat anti-rabbit IgG Alexa Fluor 488, 1:500
Blocking	5% BSA in PBS, 1 hour RT
Fixation	4% PFA, 15 min
Nuclear counterstain	DAPI, 1 µg/mL
Imaging	Confocal microscopy, 63× oil objective

Abbreviations: BSA, bovine serum albumin; PBS, phosphate-buffered saline; RT, room temperature; PFA, paraformaldehyde; DAPI, 4',6-diamidino-2-phenylindole.
