# Supplementary figures and images for: Melatonin Biosynthesis, Receptors, and the Microbiota–Tryptophan–Melatonin Axis: A Shared Dysbiosis Signature Across Cardiac Arrhythmias, Epilepsy, Malignant Proliferation, and Cognitive Trajectories
Source: Int J Mol Sci. 2026 Jan 29;27(3):1361. doi: 10.3390/ijms27031361 (PMC12898085; doi:10.3390/ijms27031361)

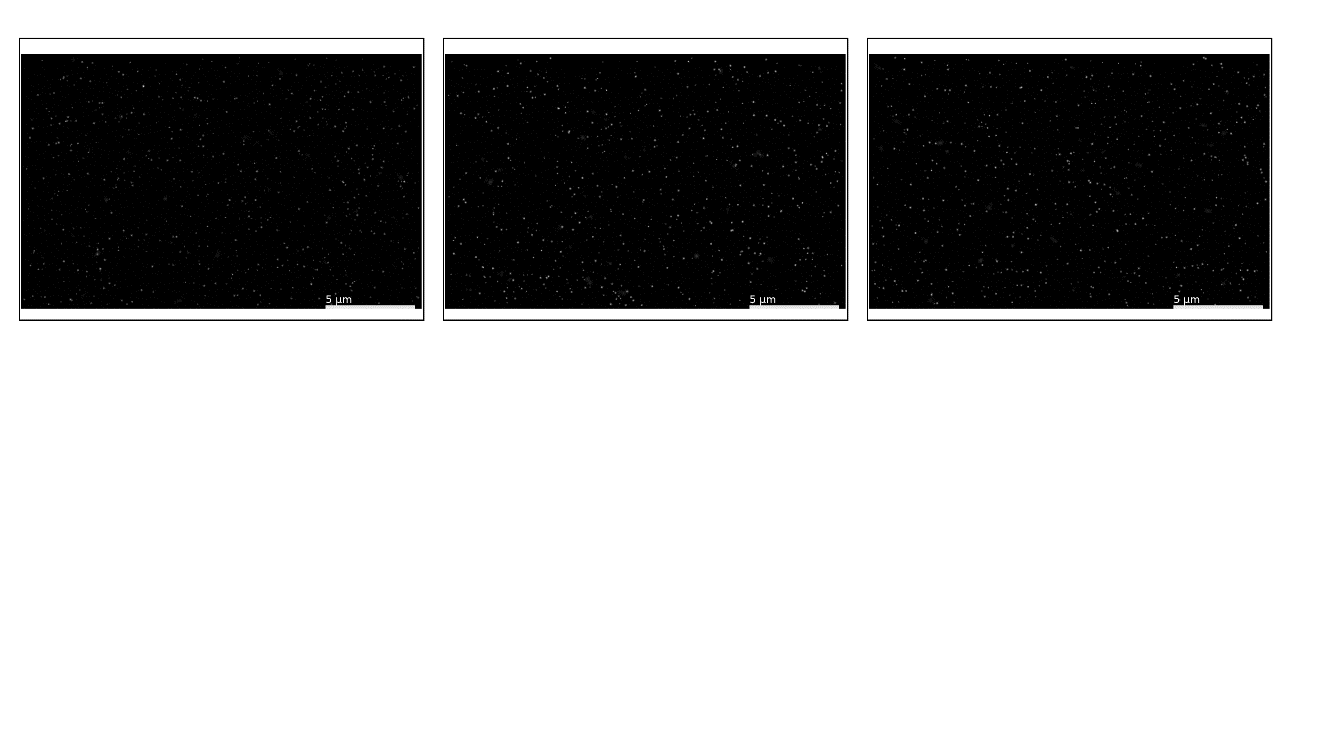

Supplement: Supplementary file 1 [file ijms-27-01361-s001.zip › Figure_S1_Isotype_Controls.png]

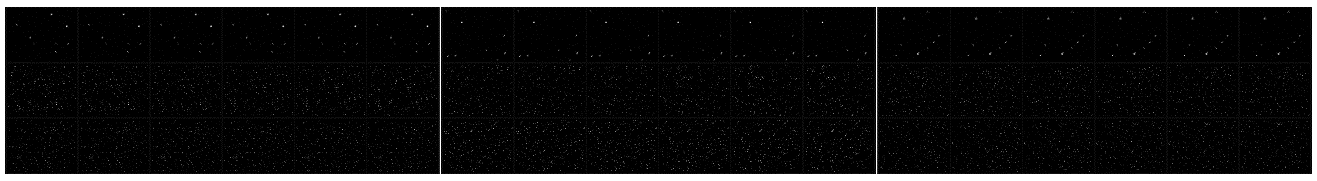

Supplement: Supplementary file 1 [file ijms-27-01361-s001.zip › Figure_S2_Peptide_Blocked_Controls.png]

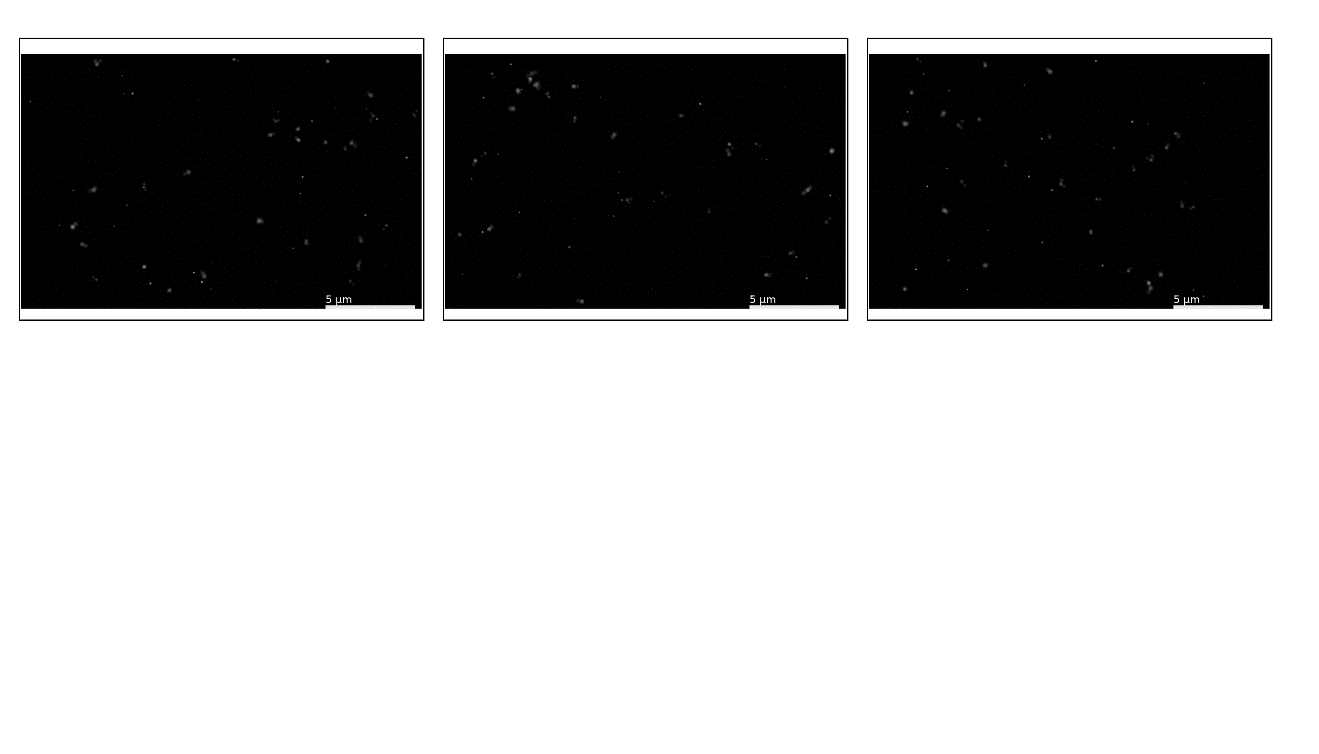

Supplement: Supplementary file 1 [file ijms-27-01361-s001.zip › Figure_S3_No_Primary_Controls.png]
